# *In vitro *vasorelaxation mechanisms of bioactive compounds extracted from *Hibiscus sabdariffa *on rat thoracic aorta

**DOI:** 10.1186/1743-7075-6-45

**Published:** 2009-11-02

**Authors:** Mamadou Sarr, Saliou Ngom, Modou O Kane, Alassane Wele, Doudou Diop, Bocar Sarr, Lamine Gueye, Ramaroson Andriantsitohaina, Aminata S Diallo

**Affiliations:** 1 Laboratoire de Physiologie Pharmaceutique, Faculté de Médecine, Pharmacie et Odontologie, Université Cheikh Anta Diop, Dakar, Sénégal; 2 Unité Mixte Internationale (UMI 3189 - Environnement, Santé et Sociétés), Université Cheikh Anta Diop, Dakar, Sénégal; 3 Laboratoire de Pharmacognosie et Molécules Naturelles Bioactives, Faculté de Pharmacie, Université Louis Pasteur, Strasbourg, France; 4 Laboratoire de Chimie Thérapeutique, Faculté de Médecine, Pharmacie et Odontologie, Université Cheikh Anta Diop, Dakar, Sénégal; 5 Laboratoire de Botanique, Institut Fondamental d'Afrique Noire, Université Cheikh Anta Diop, Dakar, Sénégal; 6 Laboratoire de Physiologie Médicale, Faculté de Médecine, Pharmacie et Odontologie, Université Cheikh Anta Diop, Dakar, Sénégal; 7 Biologie Neuro-Vasculaire Intégrée, UMR-CNRS 6214, INSERM 771, Faculté de Médecine, Université d'Angers, Angers, France

## Abstract

**Background:**

In this study, we suggested characterizing the vasodilator effects and the phytochemical characteristics of a plant with food usage also used in traditional treatment of arterial high blood pressure in Senegal.

**Methods:**

Vascular effects of crude extract of dried and powdered calyces of *Hibiscus sabdariffa *were evaluated on isolated thoracic aorta of male Wistar rats on organ chambers. The crude extract was also enriched by liquid-liquid extraction. The various cyclohexane, dichloromethane, ethyl acetate, butanol extracts obtained as well as the residual marc were subjected to Sephadex LH-20 column chromatography. The different methanolic eluate fractions were then analyzed by Thin Layer (TLC) and High Performance Liquid Chromatography (HPLC) and their vascular effects also evaluated.

**Results:**

The H. Sabdariffa crude extract induced mainly endothelium-dependent relaxant effects. The endothelium-dependent relaxations result from NOS activation and those who not dependent to endothelium from activation of smooth muscle potassium channels. The phytochemical analysis revealed the presence of phenolic acids in the ethyl acetate extract and anthocyans in the butanolic extract. The biological efficiency of the various studied extracts, in term of vasorelaxant capacity, showed that: Butanol extract > Crude extract > Residual marc > Ethyl acetate extract. These results suggest that the strong activity of the butanolic extract is essentially due to the presence of anthocyans found in its fractions 43-67.

**Conclusion:**

These results demonstrate the vasodilator potential of *hibiscus sabdariffa *and contribute to his valuation as therapeutic alternative.

## Background

Cardiovascular pathologies complications (myocardial infarction, stroke...) constitute one of the most important causes of mortality and morbidity in the world [1-3]. These complications, often facilitated by arterial high blood pressure, appear among the main causes of death in Africa. Indeed, according to World Health Organisation (W.H.O) experts, high blood pressure and hypercholesterolemia are more frequent in the developing countries than believed. Among the risk factors, except hypercholesterolemia, obesity, smoking addict and diabetes constitute the major contributing factors of these diseases[4]. A future scenario by the W.H.O. reveals a negative trend due to an increase in the rate of morbidity and mortality especially in Emerging Countries [1]. Considering the gravity and the frequency of these conditions, a search for compounds having vascular benefits is intensively pursued [5]. The interest of researchers in the whole world for these compounds encouraged us to study the healing plants of the Senegalese pharmacopoeia. Indeed, an ethnobotanical investigation led by our laboratory had listed several healing plants with antihypertensive potential among which, *Hibiscus sabdariffa *L. In the Senegalese pharmacopoeia, *H. sabdariffa *is one of the most-often used plants in the traditional treatment of high arterial blood pressure. Previous studies led by numerous groups of researchers [6-14] had already reported scientific proof of the antihypertensive effects traditionally attributed to *H. sabdariffa*. If these studies allowed demonstrating the therapeutic potential of this plant, so *in vitro *as *in vivo*, the underlying mechanisms involved as well as the phytochemical compounds responsible for these effects were not fully documented. So the objective of this study was to contribute to the understanding of such mechanisms and the discovery of bioactive substances responsible for vascular effects of *H. sabdariffa*. By combining technical preparation (extraction, enrichment, fractionation) and phytochemical characterization (TLC, HPLC) combined with biological characterization methods (organ bath), we strived to identify the phytochemical compounds and estimate their vasorelaxant effects.

## Methods

### Organic extract preparation

*H. sabdariffa *calyces was obtained from the Tilène market (Dakar). Calyces were dried during a week at room temperature, to avoid the risks of mold formation because of the relative humidity of the plant, and also to facilitate its conservation and its use during the grinding. Dried and powdered calyx (Grinder RM-100, Retsch^®^) of *Hibiscus sabdariffa *(500 g) was extracted by maceration at room temperature for 2 hours with 60% methanol. The hydroalcoholic extract was then filtered in vacuum conditions (Vacuum pump V-700, Büchi^®^) by means of the phial of Kitassato and evaporated on a rotary evaporator (Rotavapor R-210, Büchi^®^). Methanolic extract evaporation was realized during three successive days until the obtaining of a dry crude extract (136.7 g). Evaporation conditions were as follows: Temperature: +40°C; Cooling: +21°C; Rotation: 4000 tr./min. The methanolic extract, when not evaporated at once, went through those stages of separation with cyclohexane, dichloromethane, ethyl acetate and butanol to end up as an enriched extract after two hours of decantation. It is repeated as often as needed with new solvent until exhaustion (colorless organic phase). The various liquid organic extracts (cyclohexanic, 1.42 g; dichloromethanic, 2.53 g; ethyl acetate, 34.85 g; butanolic, 18.97 g and the residual marc, 79.01 g) were then washed with anhydrous sodium sulphate (Fischer^®^) to fix some residual water, and then filtered.

### Organ bath experiments

Experiments were conducted in accordance with the Guide for the Care and Use of Laboratory Animals as promulgated by the Senegalese authorities.

Male Wistar rats weighing 150-200 g were procured from a local Institute (Faculté des Sciences et Techniques, Dakar, Senegal). They were fed on standard rat feed and given free access to water. Thoracic aorta were removed from rats after anaesthesia with pentobarbital (60 mg/kg, i.p.) and cleaned of connective tissue and cut into rings (3-4 mm in length). As indicated, the endothelium was removed by rubbing the intimal surface of rings with a pair of forceps.

Rings were suspended in organ baths chambers (Panlab-TRI 202P) containing oxygenated (95% O_2_; 5% CO_2_) Krebs bicarbonate solution (mM: NaCl 119, KCl 4.7, KH_2_PO_4 _1.18, MgSO_4 _1.18, CaCl_2 _1.25, NaHCO_3 _25 and D-glucose 11, pH 7.4, 37°C) for determining changes in isometric tension. Following equilibration for 60 minutes under a resting tension of 1 g, rings were contracted with norepinephrine (1 μM) and the relaxation to acetylcholine (1 μM) was determined. After washout and a 30 min equilibration period and return to baseline, rings were contracted with cumulative concentration of norepinephrine (10^-8 ^to 10^-5 ^M), and when the contraction reached a steady state, a concentration-relaxation curve with plants extract or solvent (10^-4 ^to 10^-1^mg/mL), acetylcholine (Prolabo) or sodium nitroprusside (10^-9 ^to 3.10^-6 ^M) was constructed.

Parallel sets of experiments were performed in the presence of either the NOS inhibitor, L-NAME (300 μM), cyclooxgenase (COX) inhibitor, Indomethacin (100 μM), NO scavengers, Oxyhemoglobin (OxyHb, 10 μM) or guanylate cyclase inhibitor, Methylene blue (10 μM). Other experiments were also conducted by treatment of aortic rings, 30 min before norepinephrine contraction, with the cell-permeant SOD mimetic, manganese(III) tetrakis(1-methyl-4-pyridyl)porphyrin (MnTMPyP, 5 μM), the specific inhibitor of phosphatidyl inositol 3-kinase, Wortmanin (10 μM), the non-specific potassium channels inhibitor, Baryum chloride (BaCl_2_, 30 μM) or Glibenclamide (50 μM) which specifically blocks ATP-sensitive potassium channel.

### Phytochemical analysis

#### - Enriched extracts fractionation

Enriched extracts were fractionated by liquid Chromatography on Lipophilic Sephadex LH20^® ^(Sigma-Aldrich) according to the following protocol: 40 g of Sephadex LH20 are conditioned with methanol 20% in a glass column of 2,3 cm diameter provided with a faucet. The flow was adjusted in 32 drip/min. 1,5 g of extract were dissolved in 5 ml of methanol and deposited on the surface of the frost.

The extracts were first eluted with 120 ml of methanol/water (20:80). After the elution of 40 ml of dead volume, fractions of 200 drops are collected (fractions 1 to 11) by means of a fraction collector (Spectra/Chrom CF-1^®^). Then elution with 100 ml of methanol/water (30:70) for collection of fractions 12 to 23; 100 ml of methanol/water (40:60) for collection of fractions 24 to 35; 100 ml of methanol/water (50:50) for collection of fractions 36 to 50 and 200 ml of methanol 100% for the collection of the fractions 51 to 67. Then, the frost was washed with 250 ml of acetone/H2O mixture (50:50) to get the fractions 68 to 80. At the end of the fractionation, fractions of identical colour are combined to give fractions 1-4, 6-8, 9-15, 16-18, 19-23, 24-32, 33-42 and 43-67 for the butanolic extract. The same process of combination was applied to the other enriched extracts to get fractions 1-15, 16-20, 21-26, 27-35 and 36-67 for the ethyl acetate extract or crude extract and fractions 1-10, 11-17, 18-26, 27-40 and 41-67 for the residual marc. Each fraction was evaporated by rotary evaporator and analyzed by TLC (Silica gel 60 F254, Merck) and HPLC (Varian Pro Star).

#### - TLC-fingerprint and HPLC analysis

For the TLC analysis, extracts were dissolved in the migration solvent of the ethyl acetate/icy acetic acid/formic acid/water mixture (100:11:11:26). 10 μl of reference solutions and samples (1 mg/mL) were applied to the TLC plate. At the end of the migration, TLC Plates were dried and phytochemical compounds observed under natural light or after revelation by the NEU reagent (= 1% of diphenylboryloxyethylamine in methanol) and observation under UV light in 366 nm. Interpretation of the various chromatograms was made on the basis of those presented in Plant Drug Analysis: 6 am. Wagner, S Bladt (1996) [15]. Fluorescence can be to interpret in the following way: blue: phenolic Acids; yellow - Orange: Flavonols; Yellow - Green: Flavones.

With the aim of confirming the chemical composition of the crude extract and determining that of enriched extracts, we proceeded to an HPLC analysis. For that purpose, we used pure reference substances (chlorogenic acid, phenolic acid, delphinidin, cyanidin, etc.) of retention time and length of detection known, for the determination of the phytochemical profile of our various extracts. Extracts were examined in the following conditions: Mobile phase in gradient mode constituted by the mixture anhydrous trifluoroacetic acid 0.1% and acetonitril; debit: 1 ml/min; column C18 (EC 250/4.6 Nucleodur 100-10 C 18 ec); Diode array detector between 191 and 700 nm; injection volume: 10 μL.

### Materials

Unless otherwise indicated, drugs were purchased from Sigma Chemical Co or Aldrich (Saint Quentin-Fallavier, France). Norepinephrine (MISR CO) was a generous gift from 'Pharmacie Nationale d'Approvisionnement', Dakar, Senegal). Methanol, butanol, acetic acid, cyclohexane and dichloromethane solutions were purchased from Fischer Scientific.

### Statistical Analysis

Values are expressed as mean ± SEM. Statistical evaluation was performed with Student's t test for paired data or ANOVA. Values of p < 0.05 were considered statistically significant.

## Results

### Regulatory mechanisms of the hibiscus sabdariffa crude extract-induced relaxation

In order to characterize mechanisms involved in the relaxing effects of hibiscus sabdariffa, we conducted vascular reactivity experiments using isolated rat thoracic aortic rings treated or not with inhibitors.

#### - Influence of the endothelium

**Figure 1:**
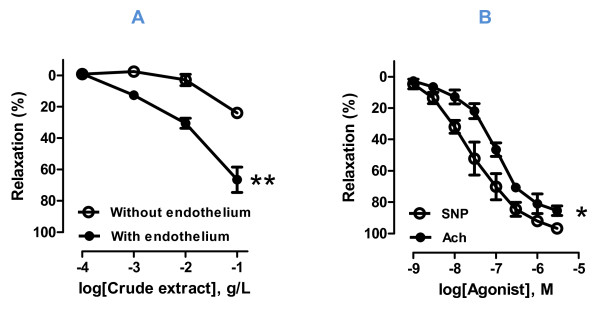
**Relaxant effect of (A) hibiscus sabdariffa calyces crude extract (10^-4 ^to 10^-1^g/l) and (B) acetylcholine or sodium nitroprusside (10^-9 ^to 3.10^-6 ^M) in aortic rings with and without endothelium precontracted with norepinephrine (10^-8 ^to 10^-6^M)**. Values are expressed as mean ± SEM of 9-12 experiments; ns: not significant; * *P *< 0.05; ** *P *< 0.01, ANOVA.

as shown in Figure 1A, *Hibiscus sabdariffa *crude extract leads to a weak relaxation (Emax: 23,93% ± 0,48) of aortic rings without endothelium. However, the observed relaxations in rings with endothelium were significantly greater (Emax: 66,57% ± 8,07). These results suggest that relaxations induced by *h. sabdariffa *crude extract are supported of both endothelium-dependent and independent mechanisms. However, the endothelium-dependent component was much more significant. In comparison with the endothelium-dependent relaxant agonist acetylcholine (Emax: 85,49% ± 3,05) or the nitric oxide donor and endothelium-independent relaxant agonist sodium nitroprusside (Emax: 96,82% ± 1,86), relaxations obtained with *h. sabdariffa *crude extract remain less pronounced (Figure 1B).

**Figure 2:**
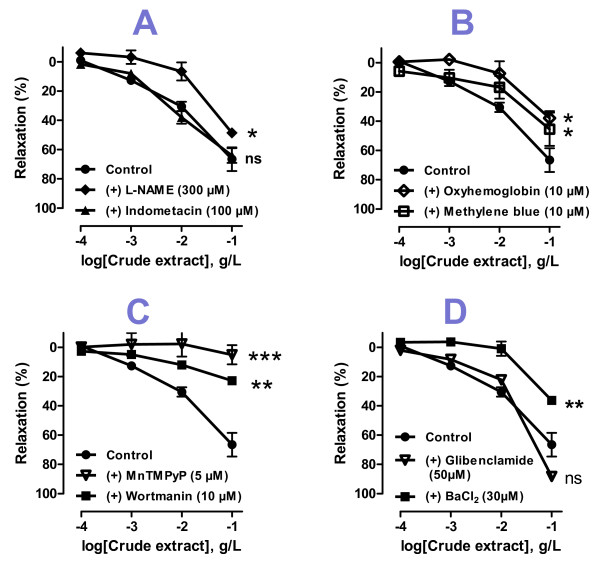
**Relaxant effect of hibiscus sabdariffa calyces crude extract (10^-4 ^to 10^-1^g/l) in aortic rings with endothelium (Control) precontracted with norepinephrine (10^-8 ^to 10^-6^M) or after treatment with inhibitors**. Values are expressed as mean ± SEM of 4-6 experiments; ns: not significant; *:*P *< 0.05, ** *P *< 0.01, in comparison to control.

As the endothelium was strongly involved in the observed relaxations, it was necessary to study the role of NO-Synthase (NOS) and Cyclooxgenase (COX), two major enzymes responsible for the release of relaxing factors in vascular beds. Using L-NAME and Indomethacin, two respective inhibitors of these enzymes, our results indicate that only NOS is activated after administration of the crude extract. Indeed, Figure 2A shows that L-NAME significantly reduced the relaxations, whereas Indomethacin does not, suggesting a possible stimulation of NO-sGC-cGMP signaling pathway by the H. sabdariffa crude extract.

Interestingly, as shown in figure 2B, the NO scavenger oxyhemoglobin and the soluble guanylate cyclase inhibitor methylene blue significantly reduce the effect of sGC activation after administration of the H. sabdariffa extract, leading to a decrease of the observed relaxations.

#### - NOS-NO-sGC pathway activation

The NO pathway was strongly involved in the relaxation induced by the crude extract of H. sabdariffa. An interesting question was how this pathway is activated. Activation of the PI3-kinase/akt pathway leads to phosphorylation of eNOS, as reported by numerous studies [16-19]. Moreover, cell-derived reactive oxygen species (ROS), when present in biological media at physiological concentrations can activate this pathway [20-22]. Our results, as shown in Figure 2C, show that wortmanin, which specifically inhibits Phosphatidyl-inositol-3-kinase (Pi3-K), as well as the SOD mimetic MnTMPyP, were found to reduce significantly the relaxations obtained with the crude extract. It is suggested that activation of the lipid kinase PI3K participate as major regulators in the NOS-initiated cascades of vasorelaxation induced by h. sabdariffa extract.

#### - Potassium channels activation

Relaxations obtained with the crude extract in vessels without endothelium, even if they are significantly lower compared with those observed in vessels with intact endothelium, led us to think a direct relaxing effect of this extract on vascular muscles. A likely mechanism is an hyperpolarization after direct activation of potassium channels. This has been verified by the non-selective inhibitor of potassium channels, barium chloride (BaCl_2_). Indeed, after treatment of vessels with this inhibitor, we observed a significant reduction of relaxations both in vessels with endothelium (Figure 2D), than in those without endothelium (data not shown). Moreover, our results also show that K^+^-ATP-dependent channels are not responsible for the endothelium-independent relaxation, as Glibenclamide, considered as a selective inhibitor of these channels does not significantly alter relaxations.

### Vascular relaxing effects of the various enriched extracts studied

**Figure 3:**
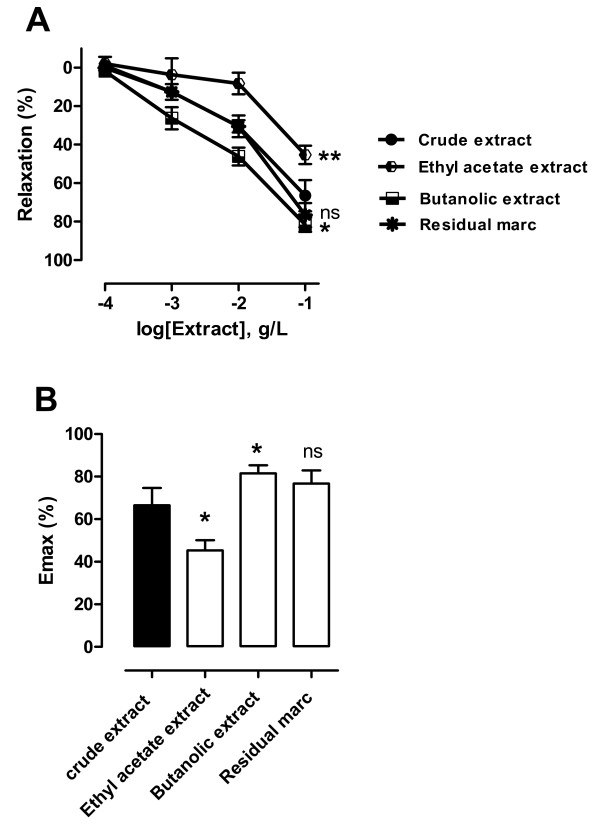
**(A) Relaxant effects of various extracts (10^-4 ^to 10^-1^g/l) of hibiscus sabdariffa calyx in aortic rings with endothelium precontracted with norepinephrine (10^-8 ^to 10^-6^M) and (B) Comparative Emax (% values in terms of vasorelaxant effects of the various enriched extracts**. Values are expressed as mean ± SEM of 6-8 experiments; ns: not significant; *: *P *< 0.05, ** *P *< 0.01, in comparison to crude extract as reference.

Since the relaxations observed with the crude extract are less than those observed with acetylcholine or sodium nitroprusside, it was necessary to make enriched extracts in order to improve the vasorelaxations. Figure 3A shows that the ethyl acetate extract causes a vasorelaxation significantly less important than the crude extract taken as reference. On the other hand, the results obtained with the butanolic extract show a vasorelaxation significantly more important than those of the crude extract. However, residual marc leads a vasorelaxation not significantly different from those of the crude extract. The biological efficiency of the various studied extracts in terms of vasorelaxant capacity was appreciated on the basis of the EC_50 _and of the maximal effect (E_max_), As indicated (Table 1 and Figure 3B), the butanolic extract presents a vasorelaxant potential more important than the other extracts. It is important to note that cyclohexanic and dichloromethanic extracts were not characterized because of their very weak return on extraction. Furthermore, these solvent allow to get rid of constituent's generally unwanted fats, chlorophylls and by-products.

**Table 1:** Comparative study of the biological efficiency in terms of vasorelaxant effects of the various enriched extracts

	**Crude extract**	**Ethyl acetate extract**	**Butanolic extract**	**Residual marc**
**EC_50 _(mg/ml)**	4,95.10^-2 ^± 1,34	4,035.10^-2 ^± 0,86	0,957.10^-2 ^± 0,6	5,579.10^-2 ^± 0,7

**E_max _(%)**	66,57 ± 8,07	34,48 ± 10,59	94,3 ± 0,97	82,44 ± 3,94

The values indicate the mean ± SEM of the EC_50 _and the E_max _obtained from 6-8 experiments.

### Phytochemical analysis of the various studied extracts

#### - TLC fingerprint and HPLC analysis of the crude extract

**Figure 4:**
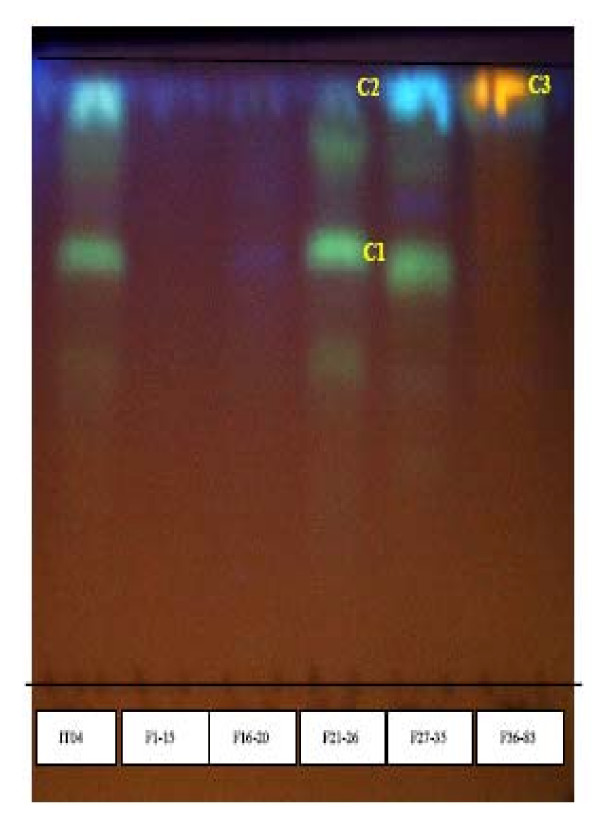
**TLC-fingerprinting of the crude extract of hibiscus sabdariffa**. **Eluent**: mixture of ethyl acetate/icy acetic acid/formic acid/water (100:11:11:26). **Detection**: under UV light in 366 nm after revelation with the reagent of NEU; **Spots**: 1 μg/μl of crude extract followed by the various fractions of elution at the same concentration: F1-15, F16-20, F21-26, F27-35 and F36-67. **Support**: Silicagel 60 F254 Merck; **Fluorescence**: blue = Phenolic Acids; yellow - Orange: Flavonols and Yellow - Green: Flavones.

**Figure 5:**
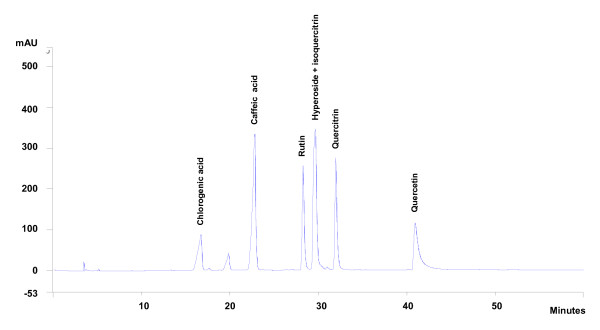
**HPLC analyze of pure substances used as references with detection to 270 nm**.

**Figure 6:**
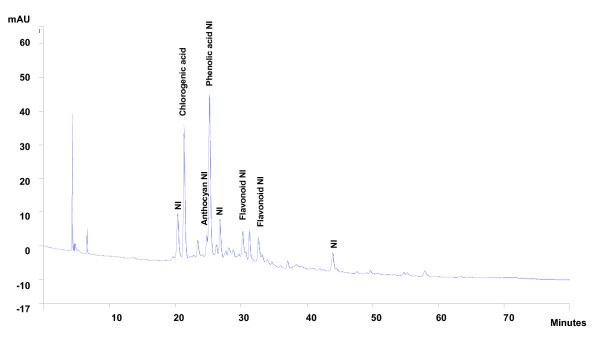
**Phytochemical profile of the crude extract after HPLC analysis and detection in 270 nm**.

To verify the presence of polyphenolic compounds whose vasorelaxant effects have already been the subject of numerous studies, we proceeded to a TLC-fingerprint analysis of the H. sabdariffa crude extract. As shown in figure 4, these compounds are indeed present in this extract. With the aim of confirming the chemical composition of the crude extract and determining that of enriched extracts, we proceeded to an HPLC analysis. To do so, we used pure reference substances (acid chlorogenic, phenolic acid, delphinidin, cyanidin, etc.), as shown in figure 5. The relative composition (expressed in percentage) of various compounds of the crude extract and their retention time are indicated in table 2. The corresponding chronograms (figure 6) show a majority of polyphenolic compounds. Results reveal a majority of phenolic and cafeic acids and the presence of flavonoids, anthocyans and not identified compounds in the crude extract.

**Table 2:** Retention time and relative composition of the crude extract after HPLC analysis and detection in the wavelength of 270 nm

**Compounds**	**Retention time (min)**	**Content in 10 mg/mL of extract (in %)**
Compound NI	20.31	7.89

Chlorogenic acid	21.30	20.18

Anthocyan NI	24.74	2.58

Phenolic acid NI	25.17	27.66

Compound NI	26.74	6.05

Flavonoid NI	30.21	4.17

Flavonoid NI	31.22	4.18

Flavonoid NI	32.59	5.35

Compound NI	43.89	4.56

NI: not identified.

#### - HPLC analysis of enriched extract

**Figure 7:**
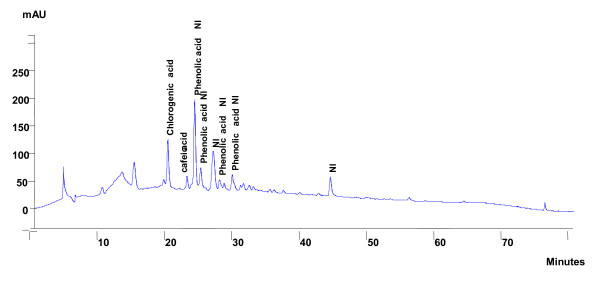
**Phytochemical profile of the ethyl acetate extract after HPLC analysis and detection in 270 nm**.

**Figure 8:**
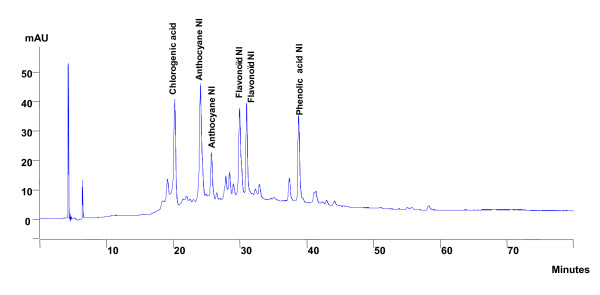
**Phytochemical profile of the butanolic extract after HPLC analysis and detection in 342 nm**.

With regards to the enriched extracts, our results show the presence of a majority of phenolic acids detected in 270 nm in the ethyl acetate extract (Figure 7, Table 3) and of anthocyans detected in 342 nm in the butanolic extract (Figure 8, table 4). Finally, the residual marc was not the object of an HPLC analysis because its vasorelaxant capacity is similar to that of the crude extract.

**Table 3:** Retention time and relative composition of the ethyl acetate extract after HPLC analysis and detection in the wavelength of 270 nm

**Compounds**	**Retention time (min)**	**Content in 10 mg/mL of extract (in %)**
Chlorogenic acid	19.66	4.46

Caffeic acid	22.52	1.97

Phenolic acid NI	23.64	4.91

Phenolic acid NI	24.54	2.38

Compound NI	26.40	4.13

Phenolic acid NI	27.33	1.58

Phenolic acid NI	29.22	2.77

Compound NI	43.79	5.03

NI: not identified.

**Table 4:** Retention time and relative composition of the butanolic extract after HPLC analysis and detection in the wavelength of 342 nm

**Compounds**	**Retention time (min)**	**Content in 10 mg/mL of extract (in %)**
Chlorogenic acid	20.16	12.08

Anthocyan NI	24.01	14.67

Anthocyan NI	25.67	5.51

Flavonoid NI	29.90	11.09

Flavonoid NI	30.93	8.37

Phenolic acid NI	38.76	7.06

NI: not identified.

#### - TLC fingerprint of the butanolic extract

**Figure 9:**
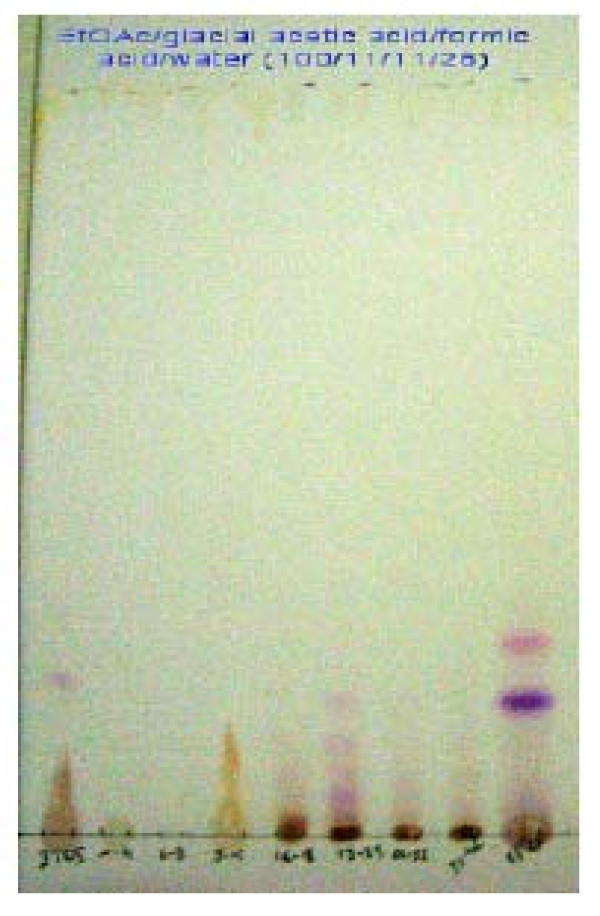
**TLC-fingerprinting of the various fractions of the butanolic extract of hibiscus sabdariffa**. **Eluent**: mixture of ethyl acetate/icy acetic acid/formic acid/water (100:11:11:26). **Detection**: in natural light without revealing; **Spots**: 1 μg/μl of butanolic extract followed by various fractions of elution at the same concentration: F1-4, F6-8, F9-15, F16-18, F19-23, F24-32, F33-42 and F43-67. **Support**: Silicagel 60 F254 Merck;

The biggest vasorelaxant capacity of the butanolic extract and its wealth in anthocyans led to us to fractionate this extract with the aim of identifying its compounds. Results (figure 9) show that only fraction 43-67 of the butanolic extract is rich in anthocyans compared with the other fractions which contain all polyphenolic compounds, in particular phenolic and chlorogenic acids or flavonoids.

## Discussion

The main results of this study demonstrate and confirm the relaxing effect of hibiscus sabdariffa extracts, especially on the isolated rat aorta. But even more interesting, they have helped characterize the possible mechanisms involved in vasorelaxation while highlighting the link between this effect and responsible phytochemical compounds.

In terms of vasorelaxation effects, analysis of our results shows that hibiscus sabdariffa effects are strongly endothelium-dependent and involve stimulation of NOS enzyme by the Pi3-K/Akt pathway. Indeed, the dominant role of the endothelium in vessel relaxation by plant polyphenols has already been demonstrated in numerous works [20,22-32]. Our results also are in agreement with data obtained from red wine polyphenolic compounds (RWPC) which activate enzymes involved in the release of endothelial relaxant factors including eNOS [27,33,34]. Moreover, our results are also in agreement with the idea that RWPC promotes the release of endothelial NO through a redox sensitive PI3/Akt pathway [22]. Finally, these results also contrast with one of our previous study [21] where it was interesting to note that the mechanism by which cognac polyphenolic compounds (CPC) enhances NO production does not involve redox system. Data obtained with this previous study demonstrate that CPC is able to directly increase NO production without affecting superoxide anions and enhances the bradykinin-induced NO production in human endothelial cells.

Our results also show a non-endothelium-dependent relaxation induced by the h. sabdariffa extract, which has not been the case for most other types of polyphenolic extracts. The likely mechanism of this non-endothelium-dependent relaxation is a direct smooth muscle activation. Indeed, H. sabdariffa as shown by our results, can also relax blood vessels without endothelium, and it was also admitted that the endothelium hyperpolarized factor (EDHF) is only marginally involved in endothelium-dependent relaxation in rat aorta [35-38]. The results of this present study strongly suggest that direct activation of smooth muscle may be caused by a membrane hyperpolarization after activation of vascular potassium channels. However, if we can exclude the role of K^+^-ATP-dependent channels, it would be interesting in the future to examine the role of K^+ ^calcium-dependent channels (IKCa, SKCa).

Numerous past studies have shown the effectiveness of Hibiscus sabdariffa in the treatment of high arterial blood pressure and other cardiovascular diseases [6-14]. If these studies, most often made from in vivo models, have already shown the biological efficiency of organic extracts of this plant, they simultaneously demonstrate the bioavailability of various constituents of hibiscus sabdariffa in biological media. The quantities of extracts to absorb will depend on the disease to treat and should be seriously studied. However, it is interesting to note the low toxicity of this plant for which toxic doses are around 2000 mg/kg [39].

On the phytochemical composition of different extracts, taking into account bibliographical knowledge [40,41], we proceeded to the enrichment of the crude extract by liquid extraction - liquid with solvents of increasing polarity to obtain various types of enriched extracts. The cyclohexanic extract allowed us to get rid of apolar constituents, generally unwanted fats, chlorophylls and by-products; The dichloromethanic extract allowed us to concentrate compounds such as terpenes, flavonoïd aglycones, coumarins, phenolic acids; the ethyl acetate extract allowed us to concentrate compounds such as flavonoïd aglycones and glycosides, phenolic acids, tannins; The butanolic extract allowed us to concentrate compounds such as flavonoïds di- and triglycerides, phenolic acids, tannins and anthocyans; the aqueous residual extract containing the rest of compounds not pulled by these solvents. The phytochemical composition of the various extracts was determined by HPLC analysis which presents numerous advantages compared with methods reported in various other works [10]. The end results, which showed a presence of polyphenols in the various examined extracts, are in line with the information given by Berhault and al. [42] as well as the works led by Lin et al. [43] and Kao et al. [44]. The only new information is the fact that the butanolic extracts, in addition to aroused polyphenols, contain mainly anthocyans.

Our study also showed that on adrenalin-precontracted isolated aortic rings, the crude hydro-alcoholic extract of *Hibiscus sabdariffa *induced a vasorelaxation. This relaxation is dose-dependent. It reaches a value of 66,57% ± 8,07 for the maximal concentration administrated i.e. 10^-1 ^mg/ml. These results concur with those of Ajay et al. [9] who showed that for a concentration of 1 mg/ml, he noted a maximal relaxation of 86% ± 4,84. However it is to note that they worked *in vitro *with a model of spontaneously hypertensive rats while we worked with normal rats.

The observed vasorelaxation is more important with the butanolic extract for which the maximal effect is about 94,3 ± 0,97% at a concentration of 10^-1 ^mg/ml in comparison with the maximal relaxation observed with the crude extract. However, the effect of the residual extract is less important than that of the butanolic and the crude extracts; and that of the ethyl acetate extract is even less. In light of these results, it appears that our study confirmed that the hydro-methanolic total extract of the dried calyces of *H. sabdariffa *possess an important vasorelaxant activity; and that the enriched butanolic extract of the dried calyces possess an vasorelaxant activity even more important. Numerous studies reported the presence and the nature of some anthocyans of *H. sabdariffa *[10,36,41,45-50]. As for the Senegalese variety of H. sabdariffa, we note especially cyanidin by-products such as cyanidin-3-monoglucoside, cyanidin-3,5-diglucoside, cyanidin-3-sambubioside [51]. After analysis, it seems that the strong vasorelaxant activity of the butanolic extract, compared with the other extracts, would be due for many to the presence of these anthocyans in his fraction 43-67. Finally, the weak quantities of extraction of this fraction did not allow us to test it on isolated rat thoracic aorta.

To the best of our knowledge, such results (the link between the vasorelaxant property and the anthocyans present in *H. Sabdariffa*) have not been reported in the literature. They thus constitute one of the originalities of our work which is to be continued, to characterize and isolate these anthocyans as well as their molecular mechanisms in the induced vasorelaxation.

## Conclusion

*Hibiscus sabdariffa *could be an alternative in the care of vascular diseases in our countries, considering its low cost and its availability. It is also necessary to emphasize the preventive role that *Hibiscus sabdariffa *could play. This is all the easier to realize as it is about a plant known, and currently used, by local populations. It would be necessary to make the populations aware of these virtues and to encourage its consumption.

## Competing interests

The authors declare that they have no competing interests.

## Authors' contributions

MS and ASD designed the study; MS, MOK and BS performed vascular studies and participated in the phytochemical characterization. AW, DD and SNG performed phytochemical experiments. MS collected the data and writes the manuscript. LG, RA and ASD corrected the manuscript and analyzed the results. All authors read and approved the final manuscript.

## References

[B1] Lazzini A, Lazzini S (2009). Cardiovascular disease: an economical perspective. Curr Pharm Des.

[B2] Luyckx VA, Yip A, Sofianou L, Jhangri GS, Mueller TF, Naicker S (2009). Cardiac function in an African dialysis population with a low prevalence of pre-existing cardiovascular disease. Ren Fail.

[B3] Meetoo D (2008). Chronic diseases: the silent global epidemic. Br J Nurs.

[B4] Misra A, Khurana L (2008). Obesity and the metabolic syndrome in developing countries. J Clin Endocrinol Metab.

[B5] Ostadal B (2009). The past, the present and the future of experimental research on myocardial ischemia and protection. Pharmacol Rep.

[B6] Mozaffari-Khosravi H, Jalali-Khanabadi BA, Afkhami-Ardekani M, Fatehi F, Noori-Shadkam M (2009). The effects of sour tea (Hibiscus sabdariffa) on hypertension in patients with type II diabetes. J Hum Hypertens.

[B7] Mojiminiyi FB, Dikko M, Muhammad BY, Ojobor PD, Ajagbonna OP, Okolo RU, Igbokwe UV, Mojiminiyi UE, Fagbemi MA, Bello SO, Anga TJ (2007). Antihypertensive effect of an aqueous extract of the calyx of Hibiscus sabdariffa. Fitoterapia.

[B8] Herrera-Arellano A, Miranda-Sanchez J, Avila-Castro P, Herrera-Alvarez S, Jimenez-Ferrer JE, Zamilpa A, Roman-Ramos R, Ponce-Monter H, Tortoriello J (2007). Clinical effects produced by a standardized herbal medicinal product of Hibiscus sabdariffa on patients with hypertension. A randomized, double-blind, lisinopril-controlled clinical trial. Planta Med.

[B9] Ajay M, Chai HJ, Mustafa AM, Gilani AH, Mustafa MR (2007). Mechanisms of the anti-hypertensive effect of Hibiscus sabdariffa L. calyces. J Ethnopharmacol.

[B10] Mahmoud BM, Ali HM, Homeida MM, Bennett JL (1994). Significant reduction in chloroquine bioavailability following coadministration with the Sudanese beverages Aradaib, Karkadi and Lemon. J Antimicrob Chemother.

[B11] Herrera-Arellano A, Flores-Romero S, Chavez-Soto MA, Tortoriello J (2004). Effectiveness and tolerability of a standardized extract from Hibiscus sabdariffa in patients with mild to moderate hypertension: a controlled and randomized clinical trial. Phytomedicine.

[B12] Odigie IP, Ettarh RR, Adigun SA (2003). Chronic administration of aqueous extract of Hibiscus sabdariffa attenuates hypertension and reverses cardiac hypertrophy in 2K-1C hypertensive rats. J Ethnopharmacol.

[B13] Onyenekwe PC, Ajani EO, Ameh DA, Gamaniel KS (1999). Antihypertensive effect of roselle (Hibiscus sabdariffa) calyx infusion in spontaneously hypertensive rats and a comparison of its toxicity with that in Wistar rats. Cell Biochem Funct.

[B14] Adegunloye BJ, Omoniyi JO, Owolabi OA, Ajagbonna OP, Sofola OA, Coker HA (1996). Mechanisms of the blood pressure lowering effect of the calyx extract of Hibiscus sabdariffa in rats. Afr J Med Med Sci.

[B15] Wagner M, Bladt S (1996). Plant Drug Analysis.

[B16] Elesgaray R, Caniffi C, Ierace DR, Jaime MF, Fellet A, Arranz C, Costa MA (2008). Signaling cascade that mediates endothelial nitric oxide synthase activation induced by atrial natriuretic peptide. Regul Pept.

[B17] Mukai Y, Shimokawa H, Matoba T, Hiroki J, Kunihiro I, Fujiki T, Takeshita A (2003). Acute vasodilator effects of HMG-CoA reductase inhibitors: involvement of PI3-kinase/Akt pathway and Kv channels. J Cardiovasc Pharmacol.

[B18] Babaei S, Teichert-Kuliszewska K, Zhang Q, Jones N, Dumont DJ, Stewart DJ (2003). Angiogenic actions of angiopoietin-1 require endothelium-derived nitric oxide. Am J Pathol.

[B19] Hurt KJ, Musicki B, Palese MA, Crone JK, Becker RE, Moriarity JL, Snyder SH, Burnett AL (2002). Akt-dependent phosphorylation of endothelial nitric-oxide synthase mediates penile erection. Proc Natl Acad Sci USA.

[B20] Anselm E, Chataigneau M, Ndiaye M, Chataigneau T, Schini-Kerth VB (2007). Grape juice causes endothelium-dependent relaxation via a redox-sensitive Src- and Akt-dependent activation of eNOS. Cardiovasc Res.

[B21] Sall Diallo A, Sarr M, Mostefai HA, Carusio N, Pricci M, Andriantsitohaina R (2008). Cognac polyphenolic compounds increase bradykinin-induced nitric oxide production in endothelial cells. Physiol Res.

[B22] Ndiaye M, Chataigneau T, Chataigneau M, Schini-Kerth VB (2004). Red wine polyphenols induce EDHF-mediated relaxations in porcine coronary arteries through the redox-sensitive activation of the PI3-kinase/Akt pathway. Br J Pharmacol.

[B23] Andriambeloson E, Kleschyov AL, Muller B, Beretz A, Stoclet JC, Andriantsitohaina R (1997). Nitric oxide production and endothelium-dependent vasorelaxation induced by wine polyphenols in rat aorta. Br J Pharmacol.

[B24] Fitzpatrick DF, Fleming RC, Bing B, Maggi DA, O'Malley RM (2000). Isolation and characterization of endothelium-dependent vasorelaxing compounds from grape seeds. J Agric Food Chem.

[B25] Andriambeloson E, Magnier C, Haan-Archipoff G, Lobstein A, Anton R, Beretz A, Stoclet JC, Andriantsitohaina R (1998). Natural dietary polyphenolic compounds cause endothelium-dependent vasorelaxation in rat thoracic aorta. J Nutr.

[B26] Andriambeloson E, Stoclet JC, Andriantsitohaina R (1999). Mechanism of endothelial nitric oxide-dependent vasorelaxation induced by wine polyphenols in rat thoracic aorta. J Cardiovasc Pharmacol.

[B27] Duarte J, Andriambeloson E, Diebolt M, Andriantsitohaina R (2004). Wine polyphenols stimulate superoxide anion production to promote calcium signaling and endothelial-dependent vasodilatation. Physiol Res.

[B28] Mendes A, Desgranges C, Cheze C, Vercauteren J, Freslon JL (2003). Vasorelaxant effects of grape polyphenols in rat isolated aorta. Possible involvement of a purinergic pathway. Fundam Clin Pharmacol.

[B29] Dell'Agli M, Busciala A, Bosisio E (2004). Vascular effects of wine polyphenols. Cardiovasc Res.

[B30] Ralay Ranaivo H, Diebolt M, Schott C, Andriantsitohaina R (2004). Polyphenolic compounds from Cognac induce vasorelaxation in vitro and decrease post-ischaemic cardiac infarction after an oral administration. Fundam Clin Pharmacol.

[B31] Olalye MT, Rocha JB (2007). Commonly used tropical medicinal plants exhibit distinct in vitro antioxidant activities against hepatotoxins in rat liver. Exp Toxicol Pathol.

[B32] Chan SL, Capdeville-Atkinson C, Atkinson J (2008). Red wine polyphenols improve endothelium-dependent dilation in rat cerebral arterioles. J Cardiovasc Pharmacol.

[B33] Martin S, Andriambeloson E, Takeda K, Andriantsitohaina R (2002). Red wine polyphenols increase calcium in bovine aortic endothelial cells: a basis to elucidate signalling pathways leading to nitric oxide production. Br J Pharmacol.

[B34] Martin S, Giannone G, Andriantsitohaina R, Martinez MC (2003). Delphinidin, an active compound of red wine, inhibits endothelial cell apoptosis via nitric oxide pathway and regulation of calcium homeostasis. Br J Pharmacol.

[B35] Bauersachs J, Popp R, Hecker M, Sauer E, Fleming I, Busse R (1996). Nitric oxide attenuates the release of endothelium-derived hyperpolarizing factor. Circulation.

[B36] Castillo C, Reyes G, Escalante B, Lopez P, Castillo EF (1997). Endothelium-dependent vasodilatation in rat aorta is mainly mediated by nitric oxide. Proc West Pharmacol Soc.

[B37] Endo K, Abiru T, Machida H, Kasuya Y, Kamata K (1995). Endothelium-derived hyperpolarizing factor does not contribute to the decrease in endothelium-dependent relaxation in the aorta of streptozotocin-induced diabetic rats. Gen Pharmacol.

[B38] Shimokawa H, Yasutake H, Fujii K, Owada MK, Nakaike R, Fukumoto Y, Takayanagi T, Nagao T, Egashira K, Fujishima M, Takeshita A (1996). The importance of the hyperpolarizing mechanism increases as the vessel size decreases in endothelium-dependent relaxations in rat mesenteric circulation. J Cardiovasc Pharmacol.

[B39] Fakeye TO, Pal A, Bawankule DU, Yadav NP, Khanuja SP (2009). Toxic effects of oral administration of extracts of dried calyx of Hibiscus sabdariffa Linn. (Malvaceae). Phytother Res.

[B40] Gonzalez-Palomares S, Estarron-Espinosa M, Gomez-Leyva JF, Andrade-Gonzalez I (2009). Effect of the temperature on the spray drying of Roselle extracts (Hibiscus sabdariffa L. Plant Foods Hum Nutr.

[B41] Segura-Carretero A, Puertas-Mejia MA, Cortacero-Ramirez S, Beltran R, Alonso-Villaverde C, Joven J, Dinelli G, Fernandez-Gutierrez A (2008). Selective extraction, separation, and identification of anthocyanins from Hibiscus sabdariffa L. using solid phase extraction-capillary electrophoresis-mass spectrometry (time-of-flight/ion trap). Electrophoresis.

[B42] Berhault J (1979). Flore illustrée du Sénégal. Edition Clairafrique Tome.

[B43] Lin HH, Huang HP, Huang CC, Chen JH, Wang CJ (2005). Hibiscus polyphenol-rich extract induces apoptosis in human gastric carcinoma cells via p53 phosphorylation and p38 MAPK/FasL cascade pathway. Mol Carcinog.

[B44] Kao ES, Hsu JD, Wang CJ, Yang SH, Cheng SY, Lee HJ (2009). Polyphenols extracted from Hibiscus sabdariffa L. inhibited lipopolysaccharide-induced inflammation by improving antioxidative conditions and regulating cyclooxygenase-2 expression. Biosci Biotechnol Biochem.

[B45] Kao ES, Tseng TH, Lee HJ, Chan KC, Wang CJ (2009). Anthocyanin extracted from Hibiscus attenuate oxidized LDL-mediated foam cell formation involving regulation of CD36 gene. Chem Biol Interact.

[B46] Mourtzinos I, Makris DP, Yannakopoulou K, Kalogeropoulos N, Michali I, Karathanos VT (2008). Thermal stability of anthocyanin extract of Hibiscus sabdariffa L. in the presence of beta-cyclodextrin. J Agric Food Chem.

[B47] Sukwattanasinit T, Burana-Osot J, Sotanaphun U (2007). Spectrophotometric method for quantitative determination of total anthocyanins and quality characteristics of roselle (Hibiscus sabdariffa). Planta Med.

[B48] Hou DX, Tong X, Terahara N, Luo D, Fujii M (2005). Delphinidin 3-sambubioside, a Hibiscus anthocyanin, induces apoptosis in human leukemia cells through reactive oxygen species-mediated mitochondrial pathway. Arch Biochem Biophys.

[B49] Frank T, Janssen M, Netzel M, Strass G, Kler A, Kriesl E, Bitsch I (2005). Pharmacokinetics of anthocyanidin-3-glycosides following consumption of Hibiscus sabdariffa L. extract. J Clin Pharmacol.

[B50] Wang CJ, Wang JM, Lin WL, Chu CY, Chou FP, Tseng TH (2000). Protective effect of Hibiscus anthocyanins against tert-butyl hydroperoxide-induced hepatic toxicity in rats. Food Chem Toxicol.

[B51] Juliani HR, Welch CR, Wu Q, Diouf B, Malainy D, Simon JE (2009). Chemistry and quality of Hibiscus (Hibiscus sabdariffa) for developing the natural-product industry in Senegal. J Food Sci.

